# Maternal Serum Concentrations of Selenium, Copper, and Zinc during Pregnancy Are Associated with Risk of Spontaneous Preterm Birth: A Case-Control Study from Malawi

**DOI:** 10.1155/2020/9435972

**Published:** 2020-04-30

**Authors:** Grace Chiudzu, Augustine T. Choko, Alfred Maluwa, Sandra Huber, Jon Odland

**Affiliations:** ^1^Department of Obstetrics and Gynecology, College of Medicine, University of Malawi, P.O. Box 30055, Lilongwe, Malawi; ^2^TB/HIV Department, Malawi Liverpool Wellcome Trust Clinical Research Programme, Blantyre, Malawi; ^3^Malawi University of Science and Technology, P .O .Box 5196, Limbe, Malawi; ^4^Department of Laboratory Medicine, University Hospital of North Norway, Sykehusveien 38, NO-9038 Tromsø, Norway; ^5^The Norwegian University of Science and Technology, Trondheim, Norway

## Abstract

Preterm birth is delivery before 37 completed weeks. A study was conducted to evaluate the association of maternal serum concentrations of selenium, copper, and zinc and preterm birth. There were 181 women in this nested case-control study, 90/181 (49.7%) term and 91/181 (50.3%) preterm pregnant women. The overall mean serum concentration of selenium was 77.0, SD 19.4 *μ*g/L; of copper was 2.50, SD 0.52 mg/L; and of zinc was 0.77, SD 0.20 mg/L with reference values of 47-142 *μ*g/L, 0.76-1.59 mg/L, and 0.59-1.11 mg/L, respectively. For preterm birth, mean serum concentration for selenium was 79.7, SD 21.6 *μ*g/L; for copper was 2.61, SD 0.57 mg/L; and for zinc was 0.81, SD 0.20 mg/L compared to that for term births: selenium (74.2; SD 16.5 *μ*g/L; *p* = 0.058), copper (2.39; SD 0.43 mg/L; *p* = 0.004), and zinc (0.73; SD 0.19 mg/L; *p* = 0.006), respectively. In an adjusted analysis, every unit increase in maternal selenium concentrations gave increased odds of being a case OR 1.01 (95% CI: 0.99; 1.03), *p* = 0.234; copper OR 1.62 (95% CI: 0.80; 3.32), *p* = 0.184; zinc OR 6.88 (95% CI: 1.25; 43.67), *p* = 0.032. Results show that there was no deficiency of selenium and zinc and there were high serum concentrations of copper in pregnancy. Preterm birth was associated with higher maternal serum concentrations of copper and zinc.

## 1. Introduction

Preterm birth occurs before 37 completed weeks (259 days) after the first day of the last menstrual period preceding the pregnancy [1]. Globally, it is estimated that 14.9 million babies are born preterm every year, with an average preterm birth rate of 11.1% per year [2]. Variations are present in different regions, ranging from 5% in western countries to 18% in some Africa countries with, Malawi being reported at 18.1% [3]. Preterm birth has adverse effects and accounts for over 75% of perinatal mortality and more than 50% of morbidity with long-term health consequences like intellectual disabilities, cerebral palsy, vision/hearing impairments, and complications of respiratory, gastrointestinal, and renal systems due to immaturity of multiple organ systems [4–6]. In addition, studies have shown significant association between preterm birth and the risk of chronic degenerative diseases such as hypertension, coronary heart disease, and type 2 diabetes mellitus during adulthood [7].

Preterm birth is a condition with many pathological processes and no precise mechanism. A common pathway involving a cellular apoptosis-transmitting inflammatory signal, senescent placental cells, and its changes in the placental membrane was hypothesised to stimulate parturition both term and preterm [8]. This common pathway is believed to be stimulated by the premature aging of the placenta caused by oxidative stress [9]. The placenta is the main source of reactive oxygen species (ROS) which induces cellular damage by acting on proteins and lipids [9]. Excessive production of ROS or impaired antioxidative defence results in a variety of pregnancy complications like preeclampsia and preterm birth [9–11]. Infections also upregulate ROS, increasing the risk of preterm birth. The micronutrients of selenium (Se), copper (Cu), and zinc (Zn) act as cofactors of antioxidant enzymes that counterbalance the oxidative stress and regulate the inflammatory response [12, 13].

Micronutrients, though required in minute quantities, are necessary inorganic constituents of human health. Their concentration status could lead to adverse pregnancy outcomes. Selenium is integrated into proteins to make selenoproteins which include glutathione peroxidase, thioredoxin reductases, and selenoprotein-P [14]. Reduced levels of maternal selenium concentrations have been shown to be associated with recurrent early pregnancy loss and preeclampsia [15–19]. Copper is an essential cofactor for numerous enzymes involved in various biological processes. The effect of maternal copper concentration is not well understood with some studies suggesting increased preterm birth with copper deficiency [20–23] and others reporting no association [24, 25]. Zinc is a crucial component of many metalloenzymes participating in protein and carbohydrate metabolism, nucleic acid synthesis, and antioxidant functions through the Cu/Zn superoxide dismutase [26]. It also assists with fetal brain development during pregnancy, as well as with being an aid to the mother during labour [27]. Changes in zinc homeostasis have been associated with various effects on pregnancy including prolonged labour, fetal growth restriction, fetal death, preeclampsia, and preterm birth [28–32].

Haematological concentrations of micronutrients are influenced by diet, lifestyle, and environmental conditions. Whether maternal concentrations of selenium, copper, and zinc during pregnancy have an effect on preterm birth in sub-Saharan Africa needs to be further determined. Subsequently, this study was conducted to evaluate the status of selenium, copper, and zinc in pregnancy and whether their concentrations are different between term and preterm pregnancies in a Malawian population. The knowledge gained on the exact role these micronutrients play in pregnancy would assist in improving nutrition in pregnancy to reduce the incidence of pregnancy complications specifically preterm birth.

## 2. Materials and Methods

This nested case control study was conducted between June 2016 and March 2017 at Kamuzu Central Hospital and Bwaila Hospital, tertiary and general hospitals, respectively. The two hospitals are public and government funded and both located within the city of Lilongwe, the capital of Malawi in the central region. Spontaneous preterm birth patients within the district can be managed at either hospital. The early preterm births (gestation of <34 weeks) are referred to Kamuzu Central Hospital because of the availability of neonatal services while the late preterm births (>34–<37weeks) are managed at Bwaila Hospital. Therefore, the two hospitals were chosen in order to capture both early and late spontaneous preterm births. Our main outcome was to determine the concentrations of selenium, copper, and zinc in term and spontaneous preterm births. The secondary outcome was to determine the association of spontaneous preterm birth and maternal serum concentrations of selenium, copper, and zinc.

The target populations were all pregnant women presenting with spontaneous preterm birth at these sites. Gestational age was based on prior-dating ultrasound. For women coming without ultrasound-dated gestation, ultrasound was done at presentation prior to recruitment, and a Ballard score was done after delivery to exclude small for gestation age babies [33]. The cases were defined as preterm pregnancies of gestation 26–<37 weeks while controls were term pregnancies of gestation 37–≤41 completed weeks. The inclusion criteria were the following: singleton pregnancy, presenting in spontaneous labour with intact membranes, and willing to participate in the study. Exclusion criteria included women having medically indicated termination of pregnancy, multiple gestations, and pregnancy complications like abruption placentae, preeclampsia, and clinical evidence of chorioamnionitis as these would cause preterm birth. Recruitment was done by a trained midwife while on duty, day or night including the weekends. Systemic random sampling was used with numbers allocated to all eligible preterm cases and all with odd numbers recruited in the study. For controls, they were recruited as they presented themselves in the labour ward matched to the living location.

Soon after the delivery process, the trained midwife proceeded to withdraw 5 mL of venous blood using a regular vacutainer technique into a royal blue top vacutainer tube (lot no. BD368380). The blood samples were immediately transported to a Baylor project laboratory within the hospital premises where centrifugation at 3000 g for 10 minutes was done. Serum was transferred into a screw-top vial and stored in the freezer at -80°C. Metal-free vessels were used throughout the process. When the required number of specimens was reached, the serum samples were shipped in the frozen state to the University Hospital of North Norway (UNN), Tromsø, Norway, for chemical analysis.

A structured questionnaire was administered in the vernacular language (Chichewa)—a local language which study participants were conversant with—by the trained midwife. Quantitative data were captured on demographic characteristics of the participants: age, gravidity, marital status, educational level, and previous history of preterm birth. Information on antenatal care package including gestation age, serology test results for human immunodeficiency virus (HIV), and syphilis was collected from the patient's health passport. Self-reported information on herbal medicine ingestion defined by the World Health Organization (WHO) as “any medicinal product based on herbs, herbal materials, herbal preparations and finished products that contain as active ingredients parts of plants, other than plant materials, or combinations thereof” and a bedside laboratory test of haemoglobin using HemoCue were documented in the questionnaire.

The chemical analyses were conducted at the Norwegian laboratory in Tromsø, using inductively coupled plasma mass spectrometry (ICP-MS) for analysis of the trace elements selenium (Se), copper (Cu), and zinc (Zn). For sample preparation, an automated liquid handler Tecan Freedom Evo 200 (Männedorf, Switzerland) equipped with an 8-channel liquid handler arm (LiHa) for conductive disposable tips, a robotic manipulator arm (RoMa) for transport of microtiter plates, and a shaker (BioShake, Quantifoil Instruments GmBH, Jena, Germany) were used. 100 *μ*L serum was diluted with a solution consisting of Milli-Q water (Millipore/Merck KGaA, Darmstadt, Germany) with 0.08% *v*/*v* Triton X-100 (Sigma/Merck KGaA, Darmstadt, Germany), 10% *v*/*v* ammonium (Honeywell Fluka, Bucharest, Romania), isopropanol (Honeywell Fluka, Bucharest, Romania), and 0.25 *μ*g/L gold (Au; Inorganic Ventures, Christiansburg, VA, USA) and mixed in the bioshaker for 2 minutes. The instrumental analysis was performed on a NEXION 300D ICP-MS system (PerkinElmer, Waltham, Massachusetts, USA) equipped with an ESI-Fast SC2DX auto sampler. An internal standard solution was introduced online via a T-piece containing 20 *μ*g/L rhodium (Rh^103^; Inorganic Ventures, Christiansburg, VA, USA). For the MS analysis, the kinetic-energy-discrimination (KED) mode was applied. Measurements were conducted in triplicates. All concentrations were obtained by the internal standard method together with a blank subtraction using the Nexion software (version 1.5). The trace element concentrations within the samples were determined based on a 3-point external matrix matched calibration curve using the ClinCal serum calibration material from Recipe (Recipe, Munich, Germany), which was diluted together with each batch of 32 samples by 1 : 100, 1 : 40, and 1 : 20. For quality assurance and quality control, two sets of ClinCheck control materials L-1 and L-2 from Recipe (Recipe, Munich, Germany) and Seronorm L-1 and L-2 (Sero, Billingstad, Norway), as well as 4 Milli-Q water blanks, were run together with each batch of samples. Diluent blanks for control of the background and instrumental carryover were also included. Additionally, the Laboratory for Analysis of Environmental Pollutants, UNN, Norway, participates successfully in the international quality control programme: Quebec Multielement External Quality Assessment Scheme (QMEQAS) organized by the Centre de toxicologie du Quebec, Quebec, Canada.

The study was reviewed and approved by the College of Medicine Research Committee (COMREC) P.05/15/1738. In addition, the Directors of Kamuzu Central and Bwaila Hospitals approved that the study can take place at their respective hospitals. Written informed consent was obtained from all study participants.

### 2.1. Data Management and Analysis

Quantitative data from the questionnaires and laboratory reports were entered into IBM SPSS version 14.0 software by a data entry clerk and verified by the principal investigator. Data cleaning was performed involving a range of consistency checks, and all outliers were rectified.

Data analysis was done using R (R Core team, 2015). A binary variable was generated for the primary outcome based on gestation: 26–<37 weeks (preterm birth as cases) coded as 1 and ≥37–41 weeks (term birth as controls) as 0. One-way frequencies were computed for categorical variables while mean and standard deviation (SD) or median and interquartile range (IQR) were computed for continuous variables that were normally distributed or skewed, respectively. Log transformation was done where necessary for positively skewed continuous variables.

Two-way associations between independent variables and the outcome variable of the preterm birth were investigated using Pearson's correlation for continuous independent variables and the chi-squared test for categorical variables. *p* values from all tests of less than 0.05 were considered statistically significant. Logistic regression models were fitted, addressing each covariate with the outcome variable. Variables were included in the multiple logistic regression model based on the best fit regression model from stepwise regression analysis using the R software based on the observed association in the unadjusted analysis at the *p* < 0.05 test level of significance. Odds ratios and associated 95% confidence intervals (CI) were computed from the logistic regression models.

## 3. Results and Discussion

There were 181 mothers with an overall mean maternal age of 25.2 years (SD: 6.1) and 181 babies participating in the present study (Table 1). The majority were married or cohabitating (173/181, 96%) and presented in the first or second pregnancy (108/181, 60%). Fifty percent had attained some primary education. Overall, 9% (16/181) gave a history of delivery of previous preterm birth while 17.7% (32/181) reported having used herbal medications in the current pregnancy. Human immunodeficiency virus and syphilis were present in 27/181 (15%) and 1/181 (0.6%), respectively.

### 3.1. Two-Way Associations with Being Preterm Birth

There were 90/181 (49.7%) term and 91/181 (50.3%) spontaneous preterm births in the present study (Table 1). Women delivering at term were older, with a mean age of 26.3 years (SD: 5.9) compared to 24.0 years (SD: 6.2), *p* = 0.011, for women who had preterm births. Women who had spontaneous preterm births were more likely to have low gravidity than those who had a term birth (*p* = 0.048). Use of herbal medications was significantly higher among women with spontaneous preterm birth (22/91, 24.2%) than among those with term birth (10/90, 11.1%), *p* = 0.035. Syphilis was significantly associated with preterm birth, *p* = 048.

Two-way analyses were also investigated for a number of trace elements (Table 2). The overall mean concentration values of selenium (77.0 *μ*g/L; SD 19.4 *μ*g/L) and zinc (0.77 mg/L; SD 0.20 mg/L) were within the normal laboratory reference values of 47-142 *μ*g/L and 0.59-1.11 mg/L, respectively. The overall mean concentration value of copper (2.50 mg/L; SD 0.52 mg/L) was above normal laboratory reference value (0.76-1.59 mg/L). All concentrations of trace elements were higher among the cases compared to the controls. Selenium concentrations were marginally increased for women with spontaneous preterm birth (mean 79.7 *μ*g/L; SD: 21.6 *μ*g/L) compared to those with term births (mean 74.2 *μ*g/L; SD: 16.5 *μ*g/L), *p* = 0.058. Copper and zinc concentrations were significantly higher for women with spontaneous preterm births than for those with term births with a mean of 2.61 mg/L (SD: 0.57 mg/L) vs. 2.39 mg/L (SD: 0.43 mg/L), *p* = 0.004, for copper and a mean of 0.81 mg/L (SD: 0.20 mg/L) vs. 0.73 mg/L (SD: 0.19 mg/L), *p* = 0.006, for zinc.

In an adjusted analysis, similar results to those for the unadjusted analysis were observed for the metals (Table 3). There was no association between a unit increase in selenium concentration and the increased odds of being a spontaneous preterm birth, OR 1.01 (95% CI: 0.99; 1.03, *p* = 0.234). Although there was an association between a unit increase in copper and the increased odds of being a spontaneous preterm birth in the unadjusted analysis (OR 2.38, CI: 1.31; 4.54, *p* = 006), this association was no longer significant in the adjusted analysis, OR 1.62 (95% CI: 0.80; 3.32, *p* = 0.184). However, there was an association between a unit increase in zinc and an increased risk for being a preterm birth in both unadjusted, OR 8.56 (95% CI: 1.83; 46.10, *p* = 009), and adjusted analyses, OR6.88 (95% CI: 1.25; 43.67, *p* = 0.032). Women who had taken herbal medication had increased odds of having a spontaneous preterm birth compared to those who did not take the herbal medication; however this was not significant, OR2.44 (95% CI:0.97, 6.52, *p* = 0.064).

The current study found that the overall serum concentrations for selenium and zinc were within normal reference values while that of copper was above normal reference values. Concentrations of selenium, copper, and zinc were higher in women who had spontaneous preterm birth compared to those who delivered at term, with copper and zinc concentrations being significant. After adjusting for other variables, increase in zinc concentration was associated with increased risk of spontaneous preterm birth.

Very little information was found in the literature on the maternal serum concentrations of the trace elements selenium, copper, and zinc in pregnancy and their associations with spontaneous preterm birth in the African region. Comparing with researches from other regions, some studies have found maternal concentrations of these trace elements to be associated with preterm birth, while others have reported no associations [20, 24, 35–39]. In this study, higher selenium concentrations were observed in the maternal serum of preterm birth, but this was not significant. This finding is similar to the study by Irwinda et al. done in Indonesia, which recruited 51 women and found no difference in maternal selenium serum concentrations between term and preterm births [35]. Contrary to these study findings, a study in Lagos, Nigeria, reported selenium deficiency of 20.4% at 14-26 weeks gestation and an 8-fold increase in risk of preterm delivery in an HIV-positive population [36]. This contrast may be due to the differences in the study population and methodology with the Nigeria study being a predominantly HIV-positive population and the samples being taken during the second trimester. Likewise, in this study, women with preterm birth showed significantly higher maternal serum concentrations of copper, but this was not associated with increased risk of being a preterm birth. This finding is consistent with findings of other studies reported in the literature [20, 24, 37]. Hao et al. collected plasma and serum at the first prenatal visit between 4 and 22 gestation weeks and found that the overall medium maternal serum copper concentrations were significantly higher for preterm births than for term births in the Chinese population [38]. Contrary to the findings of other studies in the literature, this study found that the maternal serum concentration of zinc was higher in women with preterm birth than in those delivering at term, and a unit increase of zinc had a 7-fold increased risk of having a preterm birth. A study conducted by Wang et al. in a Chinese population found significant increased concentrations of zinc in women with term births compared to women with preterm births, *p* < 0001 [32]. However, the study was prospective with samples collected during the first and second trimesters. Some studies have reported no association of maternal zinc concentrations with preterm birth [35, 39]. The difference may be due to study population and methodology used.

This study finding of increased maternal serum concentrations of selenium, copper, and zinc in women with preterm birth is not surprising. As pregnancy progresses, the fetus takes up selenium, copper, and zinc with maximum concentrations reached at the end of the third trimester when the fetal liver is mature enough to store these trace elements [39–42]. Spontaneous preterm birth reduces the pregnancy duration with resultant increased maternal serum concentrations and reduced fetal hepatic stores. Clinical research indicates that serum concentrations of selenium, copper, and zinc are higher in term infants than in preterm infants [20–23, 42–44]. Consequently, with adequate nutrition, serum concentrations of selenium, copper, and zinc would be expected to be higher in the serum of women delivering preterm as there is reduced time for the mother to pass these trace elements to the placenta and fetus. The lack of significant association of maternal serum concentrations of selenium and preterm birth may be due to the small number studied as a sample size of 600 was needed to see an effect in the present population.

The mechanism for the association of maternal serum concentration and increased risk of preterm birth could be explained due to the dual function of zinc, as an antioxidant as well as a prooxidant, and the cytotoxicity of copper. The placenta is the main source for ROS [9]. Nonetheless, the placenta is equipped with antioxidants inclusive of selenium-dependent enzymes of glutathione dismutase, thioredoxin reductases, selenoprotein-P, and copper/zinc superoxide dismutase which require the investigated trace elements of selenium, copper, and zinc [40]. However, the elevated free zinc and copper ion in women delivering preterm could have caused the generation of ROS. Free zinc ions damage mitochondria and NADPH oxidases to produce ROS [45]. Copper (I) ions are also capable of producing ROS by themselves [46].

The finding of maternal serum copper concentrations above the normal limit in both preterm and term deliveries also needs further investigation. Considering that excess copper ingestion has negative health effects on humans, the study recommends looking at the social habits, diet, and water as sources of exposure for excess copper ingestion.

Women who had taken herbal medication had almost 2.5 times increased risk of having spontaneous preterm birth compared to those who did not. It is possible that herbal medicine increased the intake of zinc and copper ion in women which resulted in increased production of ROS. Further research is required to establish this link.

Some limitations should be considered when interpreting these study results. Firstly, the sample size was relatively small which reduced the power to 34% for detecting the effect. Recruitment was also only done when the research assistants were on duty, and this may have affected the randomisation. Increased sample size and purposely recruited research assistants working in shifts to recruit day and night for the seven days of the week should be considered for future studies. Secondly, the study population diet information was not available; as such, it was difficult to ascertain the daily dietary intake of these trace elements. Thirdly, data on body mass index (BMI) was not captured in this study. This made the findings of this study not comparable with the findings by van den Broek et al. which noted the association of very low BMI with preterm birth [47]. Irrespective of the findings of no overall deficiencies of these trace elements in pregnancy and the fact that women delivering preterm babies had higher concentrations of the trace elements compared to those delivering at term, there is need to establish the causal link in preterm birth. The increased maternal serum copper concentrations in both term and preterm deliveries need further investigations considering the harmful effects of excess copper.

## 4. Conclusions

No selenium, copper, or zinc micronutrient deficiencies were found in the study population. Preterm births were associated with higher maternal serum concentrations of the micronutrients copper and zinc which play a role in prooxidation and antioxidation as well as an anti-inflammatory mechanism. Copper maternal serum concentrations were above the upper normal limit in both term and preterm birth groups. As such, further assessments are recommended to determine the excess source of copper. This study is a first assessment of these trace elements in mothers of term and preterm pregnancies in Malawian women. Further assessments in first and second trimesters with dietary information are recommended to establish local reference values as well as causal relationship.

## Figures and Tables

**Table 1:** Characteristics of study participants for term versus preterm births.

Variable	Characteristic	Total *n* = 181	Term births *n* = 90	Preterm births *n* = 91	*p* value
Maternal age (years)	Mean (SD)	25.2 (6.1)	26.32 (5.92)	24.01 (6.19)	0.011^∗^
Gestation (weeks)	Mean (SD)	35.6 (1.8)	38.00 (1.04)	33.36 (2.55)	<0.0001^∗^
Birth weight (kg)	Mean (SD)	2.6 (0.4)	3.14 (0.38)	2.12 (0.48)	<0.0001^∗^
Married/cohabitating	*N* (%)	173 (96)	87 (96.7)	86 (94.5)	0.766
Gravidity	*N* (%)				0.048^∗^
(i) 1-2		108 (60)	46 (51.1)	62 (68.1)	
(ii) 3-5		61 (34)	38 (42.2)	23 (25.3)	
(iii) ≥6		12 (6.6)	6 (6.7)	6 (6.6)	
Preterm baby before	*N* (%)	16 (9)	4 (4.4)	12 (13.2)	0.38
Education level	*N* (%)				0.31
(i) ≥college		11 (6.1)	5 (5.6)	6 (6.6)	
(ii) secondary		70 (38.7)	41 (45.6)	29 (31.9)	
(iii) primary		91 (50.3)	40 (44.4)	51 (56.0)	
(iv) none		9 (5)	4 (4.4)	5 (5.5)	
Herbal medication use	*N* (%)	32 (17.7)	10 (11.1)	22 (24.2)	0.035^∗^
HIV positive	*N* (%)	27 (15)	14 (15.6)	13 (14.3)	0.975
Syphilis positive	*N* (%)	1 (0.6)	0 (0.0)	1 (1.1)	0.018^∗^

SD: standard deviation; *N*: number; kg: kilograms. *t*-test used for continuous variables, chi-square or Fisher's exact test used for categorical variables.

**Table 2:** Two-way analysis for micronutrient concentrations.

Serum concentration	Characteristic	Lab ref values [34]	Total *n* = 181	Term births *n* = 90 (mg/L)	Preterm births *n* = 91	*p* value
Selenium (*μ*g/L)	Mean (SD)	47-142	77.0 (19.4)	74.2 (16.5)	79.7 (21.6)	0.058
Copper (mg/L)	Mean (SD)	0.76-1.59	2.50 (0.52)	2.39 (0.43)	2.61 (0.57)	0.004
Zinc (mg/L)	Mean (SD)	0.59-1.11	0.77 (0.20)	0.73 (0.19)	0.81 (0.20)	0.006

SD: standard deviation. *t*-test used for continuous variables.

**Table 3:** Unadjusted and adjusted estimates for being preterm (complete case analysis).

Variable	Characteristic	Unadjusted				Adjusted			
		OR	95% CI		*p* value	OR	95% CI		*p* value
Maternal age	Yearly increase	0.94	0.89	0.99	0.012	0.99	0.92	1.07	0.862
Herbal medication	No								
	Yes	2.55	1.16	5.98	0.024	2.44	0.97	6.52	0.064
Unit increase in selenium	Unit	1.02	1.00	1.03	0.063	1.01	0.99	1.03	0.234
Unit increase in copper	Unit	2.38	1.31	4.54	0.006	1.62	0.80	3.32	0.184
Unit increase in zinc	Unit	8.56	1.83	46.10	0.009	6.88	1.25	43.67	0.032
Gravidity	1-2	Ref							
	2-5	0.45	0.23	0.85	0.015	0.68	0.29	1.61	0.381
	≥6	0.74	0.22	2.51	0.624	1.14	0.21	6.12	0.874

OR: odds ratio; CI: confidence interval; Ref: reference. Simple and multivariable logistic regression used.

## Data Availability

The SPSS data used to support the findings of this study are included within the supplementary information file.

## Supplementary Materials

Supplementary Materials The SPSS data used to support the findings of this study are included within the supplementary information file.
